# The potential role of vildagliptin in the management and prevention of type 2 diabetes mellitus

**DOI:** 10.4103/0253-7613.40482

**Published:** 2008

**Authors:** C.K. Chakraborti

**Affiliations:** Department of Microbiology, Kanak Manjari Institute of Pharmaceutical Sciences, Rourkela, Orissa, India

**Keywords:** Diabetes mellitus, dipeptidyl peptidase-4, glucagon-like peptide-1, vildagliptin

## Abstract

Proper control of blood sugar in type 2 diabetes mellitus (T2DM) is not adequate till now in spite of use of well-planned dosage regimens containing oral hypoglycemic agents/insulin or both. Recently, the role of ‘incretins,’ particularly that of glucagon-like peptide-1 (GLP-1) in glucose homeostasis has been firmly established. The peptide (GLP-1) increases insulin secretion while decreasing that of glucagon in response to rise in plasma glucose in addition to delay of gastric emptying time, reduction of appetite, preservation of beta-cell function, and increase in beta-cell mass all of which will contribute toward lowering of blood sugar in T2DM. But the peptide hormone cannot be used orally as such because of its very short plasma half-life (2 min) and chemical nature, which needs continuous i.v. infusion or repeated s.c. or i.v. injections at short intervals. Hence, to prolong the duration of action of endogenous GLP-1, compounds have been synthesized which inhibit the enzyme dipeptidyl peptidase-4 (DPP-4), the enzyme responsible for metabolic degradation of GLP-1. One such compound is vildagliptin. In this article, an attempt has been made to compile some of the established recent advances in the therapeutic utility of vildagliptin along with a discussion about the physiological role of endogenous GLP-1 and its metabolism by DPP-4.

The hallmark of type 1 diabetes mellitus (T1DM) is selective destruction of beta-cells associated with severe or complete insulin deficiency, thereby making administration of exogenous insulin mandatory. On the other hand, type 2 diabetes mellitus (T2DM), distinguished by a deficient insulin secretion of varying degree and sometimes hyperinsulinemia with insulin resistance, is treated with oral hypoglycemic agents and/or insulin, depending on progress of the disease.[1,2]

There is evidence to show that T2DM or at least impaired glucose tolerance, is associated with decreased cognition independent of age. Therefore, the normal, age-related decline in cognitive function might be aggravated in T2DM which is associated with impaired glucose tolerance and insulin resistance.[3] In both T1DM and T2DM, hyperglycemia occurs not only due to deficiency of insulin, but also due to over activity of counter-regulatory hormones like glucagon, cortisol, growth hormone, thyroxine, and adrenaline (in stressful conditions), which cause gluconeogenesis and (except cortisol) glycogenolysis.[4–6] Both these factors increase hepatic output of glucose, thereby contributing toward development of hyperglycemia, in which glucagon plays the major role. In healthy subjects, like insulin, glucagon secretion is controlled by a variety of nutrients, neural and hormonal factors, of which glucose has a very important function. The defect in alpha-cell function that occurs in T2DM reflects deranged glucose sensing by these cells.[7] Moreover, absence of proper beta-cell suppression of alpha-cell secretion has been invoked as a mechanism that clarifies exaggerated glucagon responses, especially common in patients with deficient beta-cell secretion (T1DM and insulinopenic T2DM).[8] From these facts, it can be concluded that by using exogenous insulin and/or by reducing glucagon level, blood glucose concentration may be controlled. So far, importance has been given to the first option.

Pancreatic islet dysfunction of T2DM involves alterations in both insulin and glucagon secretion since proper concentrations of both are necessary for maintenance of glucose homeostasis.[9] Although there is ample indication that hyperglucagonemia plays a key role in the development of hyperglycemia in these patients, efforts to follow and correct this abnormality have been overshadowed by the emphasis on deficient insulin secretion and action.[7]

Type 2 diabetes mellitus is at present one of the most challenging health-care problems, which requires optimum management. Current treatment for the T2DM is often associated with inadequate control of postprandial hyperglycemia (especially with sulphonylureas, metformin, and thiazolidinediones), weight gain (sulphonylureas, meglitinides, thiazolidinediones, and insulin), and loss of efficacy over time (a problem with all current oral agents). Recent knowledge of physiological responses to meals has lead to the development of novel agents whose therapeutic actions are based on the enhancement of gastrointestinal hormone secretion and action. These agents might help to reduce some of the above-mentioned problems.[10]

## Incretins

Recently, the role of ‘incretins’ in glucose homeostasis has been firmly established. The two incretins, so far identified, are protein hormones produced by special cell of the upper and lower bowel and are called glucose-dependent insulinotropic polypeptide (GIP) and glucagon-like peptide-1 (GLP-1). They are secreted following ingestion of food and through a complex mechanism, take part in the glucose homeostasis by reducing postprandial blood glucose levels.[11] Their effect, termed as ‘incretin effect’, expresses the phenomenon of an increased insulin response following oral ingestion of glucose compared with that of i.v. administration. Out of the two incretins, it is GLP-1 which has got significant effect in this respect. GLP-1 is secreted from the L-cells present in the distal ileum and colon, in response to food rich in carbohydrates and fats. The important effects of GLP-1 are enhancement of glucose-dependent insulin secretion from the pancreas, suppression of inappropriately elevated glucagon secretion, delay of gastric emptying, reduction of appetite, preservation of beta-cell function, and increase in beta-cell mass (in animal models) all of which contribute toward reduction of blood sugar.[12] In addition to these actions, several studies have shown that GLP-1 raises beta-cell neogenesis (beta-cell mass) and differentiation, and inhibits beta-cell apoptosis.[12–15] It has been established that the progressive loss of beta-cell function and mass is an early feature of T2DM, eventually leading to insulin dependence in many patients.[1] Hence, intervening early in the course of diabetes or in the prediabetic state with beta-cell differentiation and/or apoptosis could halt progression of the disease. Because of its favorable action in this respect, GLP-1 is considered to be a promising agent in reducing the incidence of T2DM, in addition to reduction of blood sugar.[16] Mention has already been made about the physiological functions of GLP-1, of which increase in glucose-dependent insulin secretion and decrease in glucagon secretion are important.[17] Infusion of GLP-1 at pharmacological concentrations diminishes blood glucose in diabetics (T2DM) as well as nondiabetics both in fasting and after feeding.[14,18] For such blood sugar reduction, any one or both of these mechanisms may be involved. It has been demonstrated that GLP-1-induced inhibition of glucagon secretion is glucose-dependent, both in healthy persons as well as in diabetics.[7,17] But, because, hyperglycemia itself can reduce glucagon secretion, it cannot be concluded definitely that inhibition of glucagon secretion by GLP-1 is entirely mediated directly. Hence, reduction of blood sugar in T2DM (where there is hyperglycemia) by GLP-1 appears to be secondary to hyperglycemia (which increases insulin secretion and decreases glucagon release) as well as due to a direct action of GLP-1 on glucagon release which is potentiated by hyperglycemia. Because of the involvement of both the mechanisms, treatment with GLP-1 is unlikely to interfere grossly with the glucagon-mediated defense against hypoglycemia.[3] Moreover, though GLP-1 decreases glucagon secretion in hyperglycemia, it does not do so in hypoglycemia; rather it may increase it.[7] This is particularly important and beneficial, because most of the currently used antidiabetic agents posses the risk of hypoglycemia.[19] Added to this, GLP-1 does not increase insulin release at normal blood glucose concentrations and hence, does not cause hypoglycemia.[20]

Several workers have shown that there is a decrease in meal-stimulated release of GLP-1 in T2DM which may be a contributing factor for hyperglycemia in such patients.[10] Moreover, insulinotropic action of GLP-1 is preserved in T2DM patients.[15] Hence, it is rational to consider GLP-1 as a therapeutic agent in T2DM, where it can improve the pancreatic function and exert its other beneficial effects.[9,21] In fact, over the past few years, in endocrinology research, a major focus is on development of a novel therapeutic strategy for T2DM, keeping GLP-1 at the center, because of its recently found insulinotropic action.[14]

## Metabolism of GLP-1

Native GLP-1 is degraded rapidly upon i.v. or s.c. administration and is therefore not suitable for routine therapy.[12] An important regulator of the biological activity of GLP-1 is an endogenous multifunctional aminopeptidase (a serine protease) enzyme, dipeptidyl peptidase-4 (DPP-4), which inactivates (N-terminal breakdown) it rapidly in rodents and humans.[11,14,22] This enzyme splits GLP-1 at the alanine residue at position 2, which not only inactivates GLP-1, but also might change it into an antagonist at the GLP-1 receptor.[14] Due to DPP-4 action, truncated metabolites are produced most of which are not active.[23] GLP-1 has a plasma half-life of approximately 2 min before being degraded by DPP-4.[10] Because of this rapid metabolism and short half-life, it cannot be used clinically.[11,24,25] To overcome short half-life problem, continuous infusion of the peptide is required to maintain steady-state levels of active GLP-1 in plasma.[13,14,18]

## Inhibitors of GLP-1

At present two approaches have been made to prolong the duration of action of GLP-1.[26] One is the use of GLP-1 receptor agonists which maintain the physiologic effects of native GLP-1, but are resistant to the actions of DPP-4 and the other is the use of DPP-4 inhibitors which decrease the inactivation of GLP-1, thereby increasing its concentration as well as its duration of action on target tissue.[11,26–28] Several investigators have concentrated on the second approach, i.e. inhibition of DPP-4 action leading to increase in the plasma concentration of GLP-1 along with an increase in its half-life and hence, prolongation of its duration of action.[11,13,26–28] Administration of specific DPP-4 inhibitors has been found to inhibit the pathway of incretin degradation along with an increase in insulin secretion.[29] Reports have confirmed that DPP-4 inhibition as a therapeutic approach for the treatment of T2DM, resulted in a rise in glucose tolerance, insulin sensitivity, and beta-cell glucose responsiveness.[28] Because of these observations, DPP-4 inhibitors have become the subjects of increasing interest to both diabetologists and pharmaceutical industry alike. These agents have established themselves as the next member of oral antidiabetic agents, designed to lower blood glucose and, possibly, prevent the progressive decrease of glucose metabolism in prediabetics and diabetics (T2DM).[23]

The mechanism of action of DPP-4 inhibitors is different from any existing class of oral glucose-reducing agents. They regulate elevated blood glucose by triggering pancreatic insulin secretion and signaling the liver to decrease glucose production.[30] Based on their mode of action, DPP-4 inhibitors appear to be of special value in early forms of T2DM, either alone or in combination with other types of oral agents.[31]

In clinical trials, DPP-4 inhibitors have demonstrated efficacy and tolerability in T2DM, without causing weight gain or hypoglycemia.[22] Moreover, when used for more than 1 year, these agents show improved efficacy over time, which may be due to GLP-1-induced increment in the number of beta-cells.[31] Clinical studies have shown that DPP-4 inhibitors alone or in combination with commonly prescribed antidiabetic agents are an effective treatment option, especially for patients with early stage of type 2 diabetes and more severe hyperglycemia.[30,32] They have been found to be suitable for once-daily oral dosing.[30] From the above observations, it appears that GLP-1-based therapy may be a beneficial approach for the treatment of T2DM.[14]

Being a protease, enzymatic action of DPP-4 is not selective. Hence, potential hazards associated with DPP-4 inhibitors are prolongation of action of other peptide hormones, neuropeptides and chemokines metabolized by the protease and their (DPP-4 inhibitors) interaction with DPP-4 related proteases.[31] Some researchers have reported that DPP-4 inhibitors have an increased risk of infection (for nasopharyngitis and urinary tract infection) and headache.[33]

## Vildagliptin

Till date, many orally active DPP-4 inhibitors have been synthesized. Of these, vildagliptin, sitagliptin, and saxagliptin are in late stages of clinical trials.[10,30] In this article, an attempt has been made to elaborate various aspects of vildagliptin; like its chemistry, pharmacokinetic and pharmacodynamic properties, therapeutic uses, and adverse effects. Vildagliptin is a potent orally active, highly selective and reversible DPP-4 inhibitor that enhances antidiabetic actions of the incretins.[9,34,35] It is 1-[[(3-hydroxy-1-adamantyl)amino]acetyl]-2-cyano-(s)-pyrrolidine P-4.[34] It improves glycemic control by inhibiting the inactivation of GLP-1 and GIP by DPP-4. This leads to increased plasma concentration and prolonged action of these incretins in response to ingestion of nutrients, which in turn raises insulin sensitivity, reduces glucagon secretion and improves beta-cell function in a glucose dependent manner.[30,35–39] Hence, glycemic control in T2DM patients improves due to increased alpha- and beta-cell responsiveness to glucose.[19,40] In addition to inhibition of GLP-1 metabolism, vildagliptin also interferes with postreceptor signaling mechanisms of GLP-1, resulting in an improvement of alpha-cell glucose sensing in patients with T2DM.[7] The drug also sends signals to the liver to reduce glucose production.[30] In a preclinical study, it has been observed that this agent expands beta-cell mass.[17] On the contrary, it arrests obesity-related rise in beta-cell mass.[41] As a result of all these actions, it significantly decreases fasting plasma glucose and prandial glucose levels.[42] Thus, vildagliptin enhances body's own ability to control blood glucose. That is why it has been mentioned earlier that their (DPP-4 inhibitors) mechanism of action is distinct from other oral glucose-lowering agents.[30]

Several workers have demonstrated the following actions of vildagliptin in T2DM patients: it reduces glycosylated-hemoglobin [HbA(1)_c_] in T2DM patients whose blood sugar is poorly controlled even with high doses of insulin. Addition of vildagliptin to insulin therapy has been found to be associated with severe hypoglycemia.[19] During meal tolerance test, single dose of vildagliptin augments insulin secretion in T2DM patients and inhibits hepatic glucose release, leading to increased suppression of endogenous glucose production (EGP). In postprandial period, a single dose of vildagliptin lowered plasma glucose levels by enhancing arrest/inhibition of EGP.[43] Alterations in islet function secondary to increased circulating concentrations of active GLP-1 are accompanied with the decreased postprandial glycemic excursion observed in the presence of vildagliptin in T2DM patients.[44] Three phase 2 studies of vildagliptin administration for 12 weeks in T2DM patients have been demonstrated. In these studies when it was used either as monotherapy or combined with metformin, the effect of drug treatment versus placebo caused significant reductions in HbA(1)_c_.[9] Therapy for 1 year also produced significant results when compared to the placebo; vildagliptin (50 mg) lowered the prandial glucose by 43 mg/dl, fasting glucose by 20 mg/dl and HbA(1)_c_ by 1.1% in T2DM patients. It has also been reported that vildagliptin treatment might improve beta-cell function.[45] A clinically meaningful reduction in HbA(1)_c_ was maintained throughout an 1-year drug treatment period in drug-naïve patients of T2DM with both metformin and vildagliptin monotherapy.[46] These studies suggest that the greater glycemic control is due to an improvement in islet function.[9] Another study reported that vildagliptin improves glycemic control both as monotherapy and in combination with metformin for periods of ≤52 weeks in patients with T2DM. This may be due to reduced fasting and prandial glucose levels and decreased HbA(1)_c_ levels.[35] When vildagliptin and metformin are used in patients with T2DM for more than 52 weeks, beta-cell function is improved along with postmeal insulin sensitivity.[18] Initial clinical trial indicates that vildagliptin is an important oral treatment option for weight neutral, glycemic control when combined with metformin.[47]

Bosi *et al.*[48] have shown that vildagliptin is well tolerated and produces clinically meaningful, dose-related reduction in HbA(1)_c_ and fasting glucose as add on therapy in patients with type 2 diabetes inadequately controlled by metformin. Studies by Ristic and Bates[9] conclude that vildagliptin is a safe and effective new approach to targeting GLP-1 deficiencies in patients with T2DM. Kleppinger and Helms[49] have found the drug to reduce glucagon concentrations (because GLP-1 activity is increased) with little or no change in insulin levels. Results of studies by Scheen[21] and Del Prato,[50] as expected, have shown vildagliptin to improve blood glucose control of T2DM patients without inducing severe hypoglycemia and without promoting weight gain, i.e., weight neutral. Bonora[51] and Mathieu and Bollaerts[52] have reported that vildagliptin offers advantages in the treatment of overweight/obese and elderly T2DM patients, respectively.

Several workers have used different dosage regimens of vildagliptin either alone or in combination with others (metformin) and found more or less similar beneficial results. With vildagliptin doses ranging from 25 mg daily to 100 mg twice daily, some researchers have noted consistent decrease in fasting plasma glucose, 4 h postprandial glucose and HbA(1)_c_. Similar results were seen when the drug was used in combination with metformin. Several studies have shown vildagliptin to be well tolerated, which is true even after 12 weeks; however, incidences of hypoglycemia increased with longer study duration. Optimal results with minimal adverse effects were obtained with 25 mg twice daily and also 50 mg once daily doses of this agent.[49] Glycemic control with doses of 50 or 100 mg/day, was improved relative to placebo in several well-designed clinical trials of vildagliptin monotherapy in patients with T2DM.[38] Similar results were found with 50 and 100 mg q.d. doses in those patients. Vildagliptin at dosages up to 100 mg q.d. seemed to be safe and well-tolerated.[53] On the other hand, another study group has mentioned that 100 mg/day of it produces similar clinical benefits whether given as single or divided doses.[54] The drug has been found to decrease HbA(1)_c_ by 0.5-1.0% with few adverse effects and no weight gain.[17] It has also been reported that subjects with higher base line HbA(1)_c_ levels showed greater response.[39]

Vildagliptin is absorbed rapidly (median time to reach maximum concentration is 1 h) and its absolute bioavailability is 85%.[55,56] Moreover, its absorption rates are slower than the elimination rate.[56] It has a half-life of about 90 min; however, ≥50% of DPP-4 inhibition is continued for more than 10 h.[9,42] That is why it is allowed for once- or twice-daily dosing.[42] In one study oral doses of vildagliptin were administered ranging from 10 to 400 mg. The *t*_max_ (time required to achieve the maximum plasma concentration) for the parent drug was noted between 0.5 and 1.5 h after the dose. *C*_max_ (peak plasma concentration) and AUC (area under plasma concentration-time curve, 0-8 h) also increased proportionately with the increase in dose. Both onset and duration of DPP-4 inhibition were dose dependent, >90% inhibition occurred within 45 min and was maintained for ≥4 h after each dose. Glucose excursions and glycogen levels during oral glucose tolerance tests were significantly and similarly reduced after each dose of vildagliptin and insulin concentrations were significantly and similarly increased after each dose.[57]

It has been reported that there is no significant difference in exposure to vildagliptin in patients with T2DM having mild, moderate, or severe hepatic impairment; therefore, no dose adjustment of vildagliptin is required in those patients.[58] Gastrointestinal adverse effects of this agent were mild-to-moderate in intensity and occurred less frequently than with metformin.[38] The most common untoward events in patients receiving vildagliptin are headache, nasopharyngitis, cough, constipation, dizziness, and increased sweating.[42] Other possible adverse effects include hyperinsulinemic hypoglycemia and nesidioblastosis due to increased circulating concentrations of GLP-1 as has been found after gastric bypass surgery.[59]

Agents like vildagliptin, sitagliptin, etc. may occupy a key place in the overall pharmacological strategy of T2DM in near future especially if the additional beneficial effects on pancreatic beta-cells are confirmed in clinical study.[21] At the same time extensive study is required to assess the advantages of these agents over current antidiabetic therapies in T2DM patients, particularly in terms of the effects on pancreatic beta-cell restoration and potential weight loss.[15] Further studies are being carried out on vildagliptin to evaluate its long-term efficacy, safety, and tolerability in comparison with other antidiabetic agents in subjects with T2DM and results are awaited.[9,35] Lastly, long-term clinical trials are required to assess the relative risk/benefit profile of these drugs compared with the established antihyperglycemic drug classes.[47]

## Conclusion

Vildagliptin, a DPP-4 inhibitor, decreases the inactivation of GLP-1 thereby increasing its (GLP-1) concentration and duration of action on target tissues which include increase in insulin secretion, accompanied with a decrease in that of glucagon. Important vildagliptin-induced beneficial effects in T2DM include significant reduction in HbA(1)_c_ along with a reduction in fasting as well as prandial plasma glucose. Moreover, vildagliptin is an effective antihyperglycemic agent in elderly patients with T2DM. In patients with T2DM, the drug is orally effective with once daily dose and when used alone or in combination with metformin, showed improved efficacy over time (may be due to GLP-1-induced increase in beta-cell number and mass) without weight gain and hypoglycemia which are common side effects of insulin and other oral hypoglycemic agents. Side effects of the drug have been found to be minor which include mild gastrointestinal upset, headache, cough, dizziness, etc. These observations, made during several clinical trials, suggest that vildagliptin and other DPP-4 inhibitors may play an important role in the management and preservation of T2DM, particularly being valuable in preventing the development and progression of the disease, which has not been possible till now with any other antidiabetic agents. Further study in this respect along with long-term safety studies and postmarketing surveillance are necessary to add these novel drugs to the existing ones for overall management of T2DM.

## References

[1] Mudaliar S, Edelman SV (2001). Insulin therapy in type 2 diabetes. Endocrinol Metab Clin North Am.

[2] Nolte MS, Karam JH, Katzung BZ (2004). Pancreatic hormones and antidiabetic drugs. Basic and Clinical Pharmacology.

[3] Perry TA, Greig NH (2003). The glucagon-like peptides: a double-edged therapeutic sword?. Tren Pharmacol Sci.

[4] Chaudhuri SK (2004). Concise medical physiology.

[5] Tripathi KD (2004). Essentials of medical pharmacology.

[6] Vasudevan DM (2005). Textbook of Biochemistry (For Medical Students).

[7] Dunning BE, Foley JE, Ahren B (2005). Alpha cell function in health and disease: Influence of glucagon-like peptide-1. Diabetologia.

[8] Young A (2005). Inhibition of glucagon secretion. Adv Pharmacol.

[9] Ristic S, Bates PC (2006). Vildagliptin: A novel DPP-4 inhibitor with pancreatic islet enhancement activity for treatment of patients with type 2 diabetes. Drugs Today (Barc).

[10] Mudaliar S (2007). New frontiers in the management of type 2 diabetes. Indian J Med Res.

[11] Winker G (2007). Incretin enhancers, incretin mimetics: From therapeutic control to clinical application. Ory Hetil.

[12] Gallwitz B (2005a). Glucagon-like peptide-1 as a treatment option for type 2 diabetes and its role in restoring beta-cell mass. Diabetes Technol Ther.

[13] Ahren B, Schmitz O (2004). GLP-1 receptor agonists and DPP-4 inhibitors in the treatment of type 2 diabetes. Horm Metab Res.

[14] Patel SK, Goyal RK, Anand IS, Shah JS, Patel HU, Patel CN (2006). Glucagon like peptide-1: A new therapeutic target for diabetes mellitus. Indian J Pharmacol.

[15] Todd JF, Bloom SR (2007). Incretins and other peptides in the treatment of diabetes. Diabet Med.

[16] Kathleen, Dungan MD, John B (2005). Glucagon-like peptide-1 based therapies for type 2 diabetes: A focus on exenatide. Clin Diabetes.

[17] Drucker DJ, Nauck MA (2006). The incretin system: Glucagon-like peptide-1 receptor agonists and dipeptidyl peptidase-4 inhibitors in type 2 diabetes. Lancet.

[18] Ahren B (2005). New strategy in type 2 diabetes tested in clinical trials: Glucagon-like peptide-1 (GLP-1) affects basic caused of the disease. Lakartidningen.

[19] Fonseca V, Schweizer A, Albrecht D, Baron MA, Chang I, Dejager S (2007). Addition of vildagliptin to insulin improves glycaemic control in type 2 diabetes. Diabetologia.

[20] Gallwitz B (2005b). Glucagon-like peptide-1 based therapies for the treatment of type 2 diabetes mellitus. Treat Endocrinol.

[21] Scheen AJ (2007). Glucagon-like peptide-1 (GLP-1), new target for the treatment of type 2 diabetes. Rev Med Liege.

[22] Green BD, Flatt PR, Bailey CJ (2006). Inhibition of dipeptidylpeptidase IV activity as a therapy of type 2 diabetes. Expert Opin Emerg Drugs.

[23] Deacon CF, Ahren B, Holst JJ (2004). Inhibitors of dipeptidyl peptidase-4: A novel approach for the prevention and treatment of Type 2 diabetes?. Expert Opin Investig Drugs.

[24] Holst JJ (2004). Treatment of type 2 diabetes mellitus with agonists of the GLP-1 receptor or DPP-4 inhibitors. Expert Opin Emerg Drugs.

[25] Arulmozhi DK, Portha B (2006). GLP-1 based therapy for type 2 diabetes. Eur J Pharm Sci.

[26] Triplitt C, Wright A, Chiquette E (2006). Incretin mimetics and dipeptidyl peptidase-4 inhibitors: Potential new therapies for type 2 diabetes mellitus. Pharmacotherapy.

[27] Kendall DM, Kim D, Maggs D (2006). Incretin mimetics and dipeptidyl peptidase-4 inhibitors: A review of emerging therapies for type 2 diabetes. Diabetes Technol Ther.

[28] Kluz J, Adamiec R (2006). New therapeutic approach in patients with type 2 diabetes based on glucagon-like peptide-1 (GLP-1) and gastric inhibitory peptide (GIP). Postepy Hig Med Dosw (Online).

[29] Scharpe S, De Meester I (2001). Peptide truncation by dipeptidyl peptidase 4: A new pathway for drug discovery?. Verh K Acad Geneeskd Belg.

[30] Barnett A (2006). DPP-4 inhibitors and their potential role in the management of type 2 diabetes. Int J Clin Pract.

[31] Mest HJ, Mentlein R (2005). Dipeptidyl peptidase inhibitors as new drugs for the treatment of type 2 diabetes. Diabetologia.

[32] Prately RE, Salsali A (2007). Inhibition of DPP-4: A new approach for the treatment of type 2 diabetes. Curr Med Res Opin.

[33] Amori RE, Lau J, Pittas AG (2007). Efficacy and safety of incretin therapy in type 2 diabetes: Systematic review and meta-analysis. JAMA.

[34] Ahren B, Sorhede Winzell M, Burkey B, Hughes TE (2005). Beta-cell expression of a dominant-negative HNF-1 alpha compromises the ability of inhibition of dipeptidyl peptidase-4 to elicit a long-term augmentation of insulin secretion in mice. Eur J Pharmacol.

[35] Ahren B (2006). Vildagliptin: An inhibitor of dipeptidyl peptidase-4 with antidiabetic properties. Expert Opin Investig Drugs.

[36] Burkey BF, Li X, Bolognese L, Balkan B, Mone M, Russell M (2005). Acute and chronic effects of the incretin enhancer vildagliptin in insulin-resistant rats. J Pharmacol Exp Ther.

[37] Mari A, Sallas WM, He YL, Watson C, Ligueros-Saylan M, Dunning BE (2005). Vildagliptin, a dipeptidyl peptidase-4 inhibitor, improves model-assessed beta-cell function in patients with type 2 diabetes. J Clin Endocrinol Metab.

[38] Henness S, Keam SJ (2006). Vildagliptin. Drugs.

[39] Prately RE, Jauffret-Kamel S, Galbreath E, Holmes D (2006). Twelve-week monotherapy with the DPP-4 inhibitor vildagliptin improves glycemic control in subjects with type 2 diabetes. Horm Metab Res.

[40] He YL, Sabo R, Riviere GJ, Sunkara G, Leon S, Ligueros-Saylan M (2007). Effect of the novel oral dipeptidyl peptidase-4 inhibitor vildagliptin on the pharmacokinetics and pharmacodynamics of warfarin in healthy subjects. Curr Med Res Opin.

[41] Raun K, von Voss P, Gotfredsen CF, Golozoubova V, Rolin B, Knudsen LB (2007). Liraglutide, a long-acting glucagon-like peptide-1 analog, reduces body weight and food intake in obese candy-fed rats, whereas a dipeptidyl peptidase-4 inhibitor, vildagliptin, does not. Diabetes.

[42] Lauster CD, McKaveney TP, Muench SV (2007). Vildagliptin: A novel therapy for type 2 diabetes mellitus. Am J Health Syst Pharm.

[43] Balas B, Baig MR, Watson C, Dunning BE, Ligueros-Saylan M, Wang Y (2007). The dipeptidyl peptidase 4 inhibitor vildagliptin suppresses endogenous glucose production and enhances islet function after single-dose administration in type 2 diabetes patients. J Clin Endocrinol Metab.

[44] Vella A, Bock G, Giesler PD, Burton DB, Serra DB, Saylan ML (2007). Effects of dipeptidyl peptidase-4 inhibition on gastrointestinal function, meal appearance and glucose metabolism in type 2 diabetes. Diabetes.

[45] Ahren B, Pacini G, Foley JE, Schweizer A (2005). Improved meal-related beta-cell function and insulin sensitivity by the dipeptidyl peptidase-4 inhibitor vildagliptin in metformin-treated patients with type 2 diabetes over 1 yr. Diabetes Care.

[46] Schweizer A, Couturier A, Foley JE, Dejager S (2007). Comparison between vildagliptin and metformin to sustain reductions in HbA(1_c_) over 1 year in drug-naïve patients with type 2 diabetes. Diabet Med.

[47] Barnett A, Allsworth J, Jameson K, Mann R (2007). A review of the effects of antihyperglycemic agents on body weight: The potential of incretin targeted therapies. Curr Med Res Opin.

[48] Bosi E, Camisasca RP, Collober C, Rochotte E, Garber AJ (2007). Effects of vildagliptin on glucose control over 24 weeks in patients with type 2 diabetes inadequately controlled with metformin. Diabetes Care.

[49] Kleppinger EL, Helms K (2007). The role of vildagliptin in the management of type 2 diabetes mellitus. Ann Pharmacother.

[50] Del Prato S (2007). Dipeptidyl peptidase 4 inhibition and vildagliptin therapy for type 2 diabetes. Int J Clin Pract Suppl.

[51] Bonora E (2007). Antidiabetic medications in overweight/obese patients with type 2 diabetes: Drawbacks of current drugs and potential advantages of incretin-based treatment on body weight. Int J Clin Pract Suppl.

[52] Mathieu C, Bollaerts K (2007). Antihyperglycaemic therapy in elderly patients with type 2 diabetes: Potential role of incretin mimetics and DPP-4 inhibitors. Int J Clin Pract Suppl.

[53] Ristic S, Byiers S, Foley J, Holmes D (2005). Improved glycemic control with dipeptidyl peptidase-4 inhibition in patients with type 2 diabetes: Vildagliptin (LAF 237) dose response. Diabetes Obes Metab.

[54] Pi-Sunyer FX, Schweizer A, Mills D, Dejager S (2007). Efficacy and tolerability of vildagliptin monotherapy in drug-naïve patients with type 2 diabetes. Diabetes Res Clin Pract.

[55] He YL, Serra D, Wang Y, Campestrini J, Riviere GJ, Deacon CF (2007). Pharmacokinetics and pharmacodynamics of vildagliptin in patients with type 2 diabetes mellitus. Clin Pharmacokinet.

[56] He YL, Sadler BM, Sabo R, Balez S, Wang Y, Campestrini J (2007). The absolute oral bioavailability and population-based pharmacokinetic modelling of a novel dipeptidylpeptidase-4 inhibitor, vildagliptin, in healthy volunteers. Clin Pharmacokinet.

[57] He YL, Wang Y, Bullock JM, Deacon CF, Holst JJ, Dunning BE (2007). Pharmacodynamics of vildagliptin in patients with type 2 diabetes during OGTT. J Clin Pharmacol.

[58] He YL, Sabo R, Campestrini J, Wang Y, Ligueros-Saylan M, Lasseter KC (2007). The influence of hepatic impairment on the pharmacokinetics of the dipeptidyl peptidase-4 (DPP-4) inhibitor vildagliptin. Eur J Clin Pharmacol.

[59] Riddle MC, Drucker DJ (2006). Emerging therapies mimicking the effects of amylin and glucagon-like peptide-1. Diabetes Care.

